# Optogenetics meets physiology

**DOI:** 10.1007/s00424-023-02887-9

**Published:** 2023-12-04

**Authors:** Sophia Ohnemus, Johannes Vierock, Franziska Schneider-Warme

**Affiliations:** 1 Institute for Experimental Cardiovascular Medicine, University Heart Center Freiburg-Bad Krozingen, Faculty of Medicine, University of Freiburg, Freiburg, Germany; 2 Spemann Graduate School of Biology and Medicine (SGBM), University of Freiburg, Freiburg, Germany; 3 Faculty of Mathematics and Physics, University of Freiburg, Freiburg, Germany; 4 Neuroscience Research Centre, Charité Berlin, Berlin, Germany

According to its classic definition, optogenetics is a method combining genetic and optical technologies to observe and/or manipulate cell-type-specific behaviour in intact biological tissues, often in living animals. While early optogenetic studies go back to the late twentieth century [14, 25, 26, 35], the optogenetic revolution occurred shortly after the millennium, triggered by the discovery of directly light-gated ion channels, including channelrhodopsin-2 (ChR2) [27]. ChR2 offered researchers the possibility of depolarising defined subsets of excitable cells upon light stimulation, used for spatially-defined induction of action potentials with millisecond precision [2, 4].

The contemporary repertoire of optogenetic tools is broad, including, but not limited to, light-activated ion channels and pumps, light-driven enzymes and G-protein-coupled receptors, light-induced dimerisers to control protein interactions and transcription, and last but not least, bioluminescent and fluorescent sensors of cell states and behaviour, such as genetically encoded indicators for Ca^2+^, voltage, and pH [6, 31]. Optogenetic approaches are now widely used to tackle open research questions in physiology, based on ground-breaking developments in the strategies for genetic delivery to the cell types of interest, including viral delivery and cell transplantation, and optical technologies for light stimulation and observation. Accordingly, optogenetic research has now extended beyond deciphering cell-type-specific contributions to neural network activity in the central nervous system to the study of many essential organs, including the cardiovascular system, the stomach and intestinal tract, the kidney, the pancreas, and the reproductive system. Besides its wide array of applications in basic research in physiology, the translational potential of optogenetics is immense, with proposed clinical applications ranging from optical deep brain stimulation for the treatment of Parkinson’s disease to optical termination of atrial and ventricular arrhythmias to optogenetic cochlear implants and vision restoration, the latter now being assessed in the first clinical trials [33]. In the following, we will shortly discuss recent developments in the field of optogenetics, highlighting publications in the current special issue on next-generation optogenetics (see Fig. 1).

**Fig. 1:**
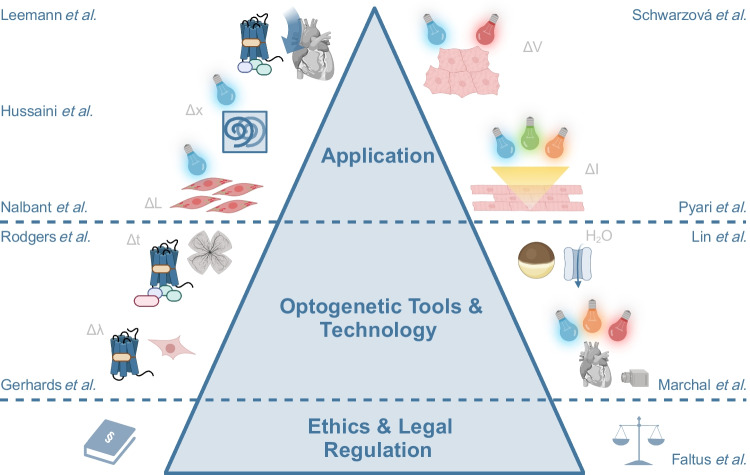
Overview of research presented in the special issue on “Recent advances in optogenetic tool design and application”

## The new kids on the block—novel tools for optophysiology research

Two decades after the identification of ChR2, the optogenetic toolbox continues to evolve, propelled by a series of unexpected discoveries of new photoreceptor classes in nature [6]. The new generation of tools holds the promise of light control that more faithfully emulates physiological processes, is less invasive, and provides compatibility with already existing sensors and actuators. The recent discovery of light-gated potassium channels (KCR) enables neuronal and cardiac inhibition through low-light hyperpolarisation [10, 38]. Molecular engineering and genetic mining of natural photoreceptor systems in diverse animal species provide a growing variety of light-controlled G-protein-coupled receptors—often described as Opto-GPCR—that allow direct control of intracellular signalling cascades [21]. Finally, the discovery of new red-shifted rhodopsins with protein absorption partly beyond 700 nm, directly coupled to enzymes or ion channel domains, promises future optogenetic manipulation deep within tissue and improved optical multiplexing with existing tools [3, 32]. However, the necessary molecular understanding of these new classes of photoreceptors is only beginning to emerge. Notably, in addition to tools building on natural and engineered photoreceptor proteins, there is an increasing number of nucleic acid-based tools for manipulation and reporting of cellular activity [5, 16, 29], further extending the possibilities of quantitative cell research.

In this special issue, Lin et al. explore the potential of recently discovered KCR in studying water transport through aquaporins in *Xenopus* oocytes. They show that the ion gradients generated by activation of anion channels (ACR), together with either sodium-selective channels (NaCR) or KCR, can be used to induce opposing osmotic gradients, which in turn cause water influx or efflux, respectively [19]. The authors suggest using this approach towards interfering with water homeostasis, which could be of interest to various organ systems, for example, for studying molecular mechanisms underlying urine concentration and body-water homeostasis in the kidney [17]. In another study, Rodgers et al. explore two optimisation strategies to accelerate the dynamics of heterologously expressed human rod opsin. They demonstrate that the lifetime of the rod opsin photoresponse can be reduced by using covalently tethered, phosphorylation-independent arrestin or by the introduction of opsin mutations close to the retinal Schiff base that favour hydrolysis of the chromophore. Furthermore, they assess the potential of their approach in an optogenetic application in degenerated rd1 retinas, validating that an improved temporal resolution can be achieved with a mutant rod opsin [23]. Finally, Gerhards et al. explore a new method of red-shifting the protein absorption that can be generalised to any all-trans retinal binding opsin. Co-expression of the enzyme Cyp27c1 converts the chromophore vitamin A1 to A2, which has higher sensitivity for light of longer wavelengths and increases the red light sensitivity of the channelrhopdopsins ChR2 and ReaChR. Notably, overexpression of Cyp27c1 in ChR-expressing HEK cells leads to a larger shift in spectral sensitivity than simply incubating cells with vitamin A2 [9].

## Optical dissection of signalling networks

Dissecting cell-type-specific contributions to network activity and organ function remains a central theme in optogenetic research. This increasingly takes into account the heterocellular nature of various organ systems [20], and the cross-talk between excitable and non-excitable cells. Optogenetic approaches, however, are not limited to the study of intercellular interactions, but can also shed light on intracellular signalling pathways [36]. We now have access to a wide array of tools for the optical manipulation and visualisation of signalling processes at key signalling hubs, e.g., by targeting transmembrane receptors and diverse enzymes involved in the production and degradation of signalling molecules, for example, second messengers.

In the issue on next-generation optogenetics, Leemann et al. comprehensively review optogenetic studies into cardiac signalling pathways beyond the optical modulation of membrane potential. Specifically, they highlight the potential of Opto-GPCR for dissecting the contribution of individual receptor subtypes to G-protein signalling, and ultimately to cardiac function, both in the healthy and diseased heart [18]. Focusing on mechanics rather than frequently studied electrical interactions, Nalbant et al. review the currently available optogenetic and photochemical methods to investigate signalling networks involved in the contraction of non-muscle cells. Next to summarising recent findings in signalling networks and cytoskeletal components that control subcellular contraction patterns, they provide an overview of light-based methods for perturbation and readout that can be combined to identify causal relations within complex cell contraction networks [28].

## Recent advances in optical technologies for whole-organ optical stimulation and imaging

In parallel to the development of new molecular tools and methods to target photoreceptor proteins to the cells of interest, optical technologies are also constantly evolving to fully serve the envisioned experiments [6]. This includes the implementation of patterned illumination for one- and two-photon excitation and all-optical approaches combining optical stimulation experiments with fluorescent imaging of cell, tissue, and organ activity. In order to enable the implementation of optogenetic studies on animal physiology and behaviour in vivo, wireless systems for minimally invasive, precise, and remotely controlled illumination and read-out have been recently developed or refined [36]. Notable examples include battery-free, lightweight optofluidic devices for combined pharmacology and optogenetics [40], and miniaturised, wireless platforms for closed-loop optogenetics [13].

Recently, Marchal et al. presented a perspective on the latest technological breakthroughs facilitating optical control and observation of cardiac electrophysiology [22]. A significant challenge in all-optical methods arises from crosstalk, caused by the overlap of the excitation and emission spectra of commonly used optogenetic proteins and/or fluorescent dyes. To tackle this issue, the authors propose an innovative approach that enables the optogenetic manipulation of cardiac electrophysiology with simultaneous monitoring of transmembrane voltage and intracellular calcium levels, establishing the basis for a multimodal investigation of whole-heart activity [22].

## Exploring the applications of optogenetics in cardiac tissue

For more than a decade, researchers have adopted optogenetic approaches developed in the neurosciences for basic cardiac research, as recently reviewed [7]. All major cell types found in the heart have now been optogenetically targeted, helping to dissect their individual contributions to whole-heart electrophysiology and contractile function [39]. One recent development includes the use of sub-threshold illumination to optically shape action potentials and spatial excitation patterns, aiming to pin down common principles underlying successful arrhythmia termination and exploiting the potential of optical defibrillation at low light levels [1, 12, 24].

The concept of sub-threshold illumination is further developed by Hussaini et al. in this issue, assessing its potential to control spiral waves in a 2D in-silico model of murine ventricles. They demonstrate that periodic low light stimulation at fixed frequency (open-loop pacing), as well as a voltage-dependent optical stimulation based on a simulated measuring electrode (closed-loop pacing), may be used to terminate arrhythmic behaviour [11]. In another article of the collection, Schwarzová et al. investigate the effects of the optogenetic tool BiPOLES in engineered heart tissue. BiPOLES is a fusion protein combining a blue light-activated ACR with a red light-activated cation-conducting ChR (CCR) [37]. While ACR is typically used for optogenetic inhibition, the study by Schwarzová et al. highlights that ACR can excite human stem cell-derived cardiomyocytes, in line with previous results demonstrating depolarising effects of ACR in adult myocytes [15]. Thus, not only pulsed activation of CCR but also of ACR could be used to optically pace engineered heart tissue, while prolonged activation of ACR reversibly silences cardiac contractility [34]. Extending these results, the in silico study by Pyari et al. analyses the effect of light attenuation on CCR or ACR-expressing human ventricular cardiomyocytes in tissue. While sustained illumination may sufficiently activate either CCR or ACR for suppressing cardiac activity at the tissue surface, myocytes in deeper tissue will receive lower light intensities insufficient to block excitation. The authors show that not only increasing the level of ChR expression but also graded channel expression, counter-balancing the effects of light attenuation, expand the optical suppression depth, and ensure synchronised excitation across tissue layers upon short light pulses [30].

## Ethical and legal aspects of optogenetic studies

The vast opportunities for using optogenetics in basic and translational research also come with the requirement to adhere to both ethical standards and legal regulations. In this issue, Faltus et al. comprehensively discuss the ethical and legal aspects of neuronal optogenetics. Specifically, they outline open ethical questions regarding optogenetic experiments using human brain organoids and experimental animals, and those questions arising when aiming to translate optogenetic technology to the clinics. They further highlight the multiple layers of legal requirements for optogenetic translation within the European Union, considering the genetic engineering, pharmaceutical, medical device, and patent laws, and the requirement of informed consent of potential future patients that would be receiving optogenetic treatments. In the optimal scenario, research into both ethical and legal aspects of optogenetics would go hand-in-hand with biomedical research to prevent hurdles in the way of optogenetic translation [8].

## Data Availability

Not applicable.
